# Venous reconstruction using a Y‐shaped saphenous vein in kidney transplantation: A report of three cases

**DOI:** 10.1002/iju5.12266

**Published:** 2021-02-16

**Authors:** Yuki Miyauchi, Terutaka Noda, Noriyoshi Miura, Tadahiko Kikugawa, Takashi Saika

**Affiliations:** ^1^ Department of Urology Ehime University Graduate School of Medicine Toon Ehime Japan

**Keywords:** kidney transplantation, living donor, major saphenous vein, vascular reconstruction, Y‐shaped reconstruction

## Abstract

**Introduction:**

Transplantation, especially, of the right kidney may be difficult to properly choose the main drainage vein due to abundance of renal veins with the thin wall and the small diameter. Therefore, we report three cases, wherein anastomosis‐related complications may be avoided by using a reconstructed Y‐shaped major saphenous vein graft.

**Case presentation:**

The first case was a case of congestion when anastomosed with a trifurcated renal vein which ligated branch. The second case was a case of donated kidney with three renal veins, which were all short, small, and thin‐walled. The third case was a case of donated kidney with four renal veins. Two of them were unused, though the other two veins were short and thin‐walled with equal diameters. In all of three cases, renal veins were anastomosed with Y‐shaped saphenous vein graft.

**Conclusion:**

Y‐shaped saphenous vein graft is possibly effective for such reconstructions as it may prevent anastomosis‐related complications.

**Abbreviations & Acronyms**
eGFR: estimated glomerular filtration rate
SVG: saphenous vein graft

**Keynote message**

Renal veins of donated right kidney are often short and thin walled. It may be difficult to select one as the main drainage vein out of multiple veins. The major saphenous vein that was procured was reconstructed into a Y‐shape and anastomosed to two renal veins.

## Introduction

Venous reconstruction is sometimes required in kidney transplantation, especially for the right kidney as the renal vein is thin and short. However, if there are multiple renal veins with equal diameters, it may not be possible to select one as the main drainage vein. We hence report three cases wherein reconstruction of two renal veins was performed using a major Y‐shaped saphenous vein in living‐donor kidney transplantations.

## Case presentation

All three cases received right kidney donations as the renal function and volume of the right kidney were inferior to those of the left kidney. The saphenous vein is procured by incising the femoral region on the same side of the transplant iliac fossa when necessary. Each patient's characteristics and clinical results were summarized in Table 1. All of these recipients had no other potential donor, and they had informed consent for the procuration of major saphenous vein and the risk of vascular accident. After operation, none of them was administered with antithrombotic agent.

**Table 1:** Patient’s characteristics and results

	Recipient age (years)	Recipient sex	Donor age (years)	Donor sex	Precured site	Site of transplanted iliac fossa	ABO compatibility	Total ischemic time (min)	Arterial reconstruction	Immuno‐suppressant agents	Delayed graft function	Postoperative hospital stay (days)	eGFR at discharge (mL/min/1.73m^2^)	Current eGFR (mL/min/1.73m^2^)
Case 1	50	Male	78	Female	Right	Right	Incompatible	288	+	Tacrolimus, mycophenolate mofetil, methylprednisolone, basiliximab, rituximab	None	22	34.4	32.4
Case 2	62	Male	62	Female	Right	Right	Incompatible	314	+	Tacrolimus, mycophenolate mofetil, methylprednisolone, basiliximab, rituximab	None	19	22.9	32.1
Case 3	41	Male	72	Male	Right	Right	Incompatible	144	−	Tacrolimus, mycophenolate mofetil, methylprednisolone, basiliximab, rituximab	None	21	33.1	34.4

### Case 1

**Fig. 1:**
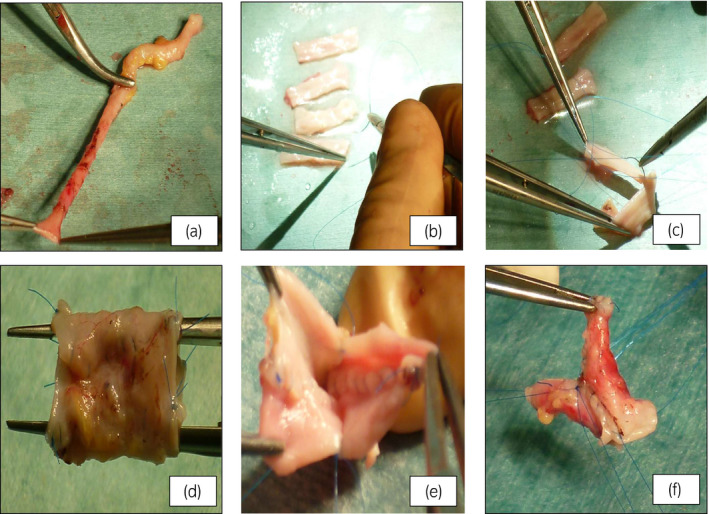
The procedure of making cylindrical shape SVG. (a) The saphenous vein ipsilateral to the transplant iliac fossa was procured. (b) The procured saphenous vein was detubularized, and split into four. (c) The specimens continuously were sutured with each with a 6‐0 proline. (d) All four specimens were sutured with each other, and a cylindrical SVG was created. (e) The procedure of making Y‐shaped SVG. Half the suture of the cylindrical SVG was removed. (f) The two vein walls were each reformed into a cylindrical shape to form a Y‐shape.

**Fig. 2:**
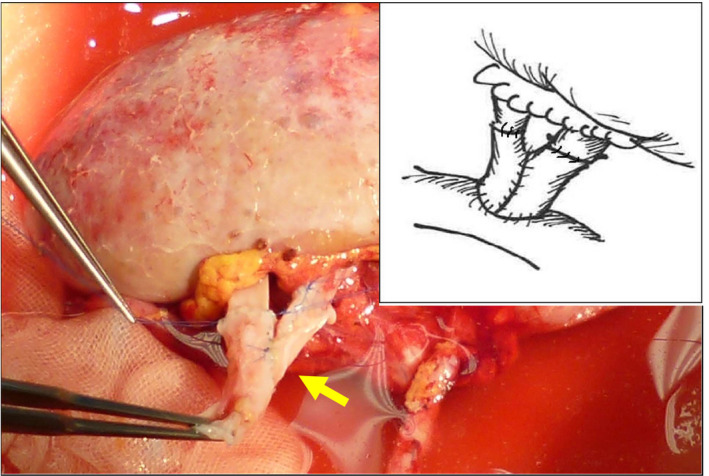
Two renal veins were anastomosed with the Y‐shaped SVG (arrow).

A 50‐year‐old male received a kidney from his mother. Since the renal vein in the donated kidney was trigeminal, it was made longer by ligating two branches. The kidney was transplanted into the right iliac fossa. During transplantation, the renal vein was thin and short, resulting in an out‐flow block, congestion, and increased hemorrhage from the transplanted kidney. Therefore, the vein was stretched using SVG after perfusing the transplanted kidney. The procured saphenous vein was detubularized (Fig. 1a), split into four sections (Fig. 1b), and continuously sutured with each other with a 6‐0 proline into a cylindrical shape (Fig. 1c) to create SVG (Fig. 1d). The SVG was anastomosed with the renal vein and blood flow was resumed, although the out‐flow block remained. Half of the cylindrical SVG suture was removed (Fig. 1e), and the two vein walls were each cylindrically reformed to form a Y‐shape (Fig. 1f). The renal vein and one of the ligated branches were anastomosed with the Y‐shaped SVG (Fig. 2). This led to improvement in renal congestion.

### Case 2

**Fig. 3:**
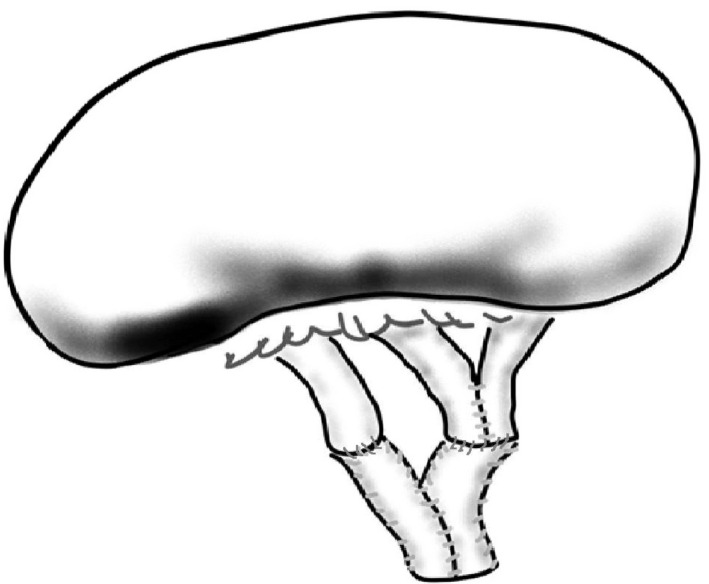
Two renal veins, anastomosed from side to side, were reconstructed into common channel. Then, another renal vein and this reconstructed vein were anastomosed with a Y‐shaped SVG.

A 62‐year‐old male received a kidney from his wife. There were three renal arteries and three renal veins in the donated kidney. The two renal arteries in the upper pole, which were anastomosed from side to side, were reconstructed into a common channel and anastomosed with the external iliac artery. One renal artery in the lower pole was anastomosed with the inferior epigastric artery. All three veins were short, equally sized, and thin‐walled. Therefore, the two renal veins, anastomosed from side to side, were reconstructed into a common channel. Then, another renal vein and the reconstructed vein were anastomosed with a Y‐shaped SVG (Fig. 3).

### Case 3

A 41‐year‐old male received a kidney from his father. There were four renal veins in the donated kidney, which were all very short, thin‐walled, and equally sized. We expected venous anastomosis to be stable if Y‐shaped SVG was used. Most cranial and caudal veins were unused, and the two central veins were anastomosed with the Y‐shaped SVG.

## Discussion

The left kidney is usually chosen for procuration since the length of the left renal vein is long. However, since the residual renal function of the donor must be optimal, the kidney with inferior function or that with anomalies such as stones or cysts may be procured, and therefore the right kidney may be donated. The right renal vein is short, has multiple renal veins, and is thin‐walled, meaning that transplant surgery may often be difficult. When the length of the renal vein is insufficient, the branches are ligated and cut, and one vein is lengthened to perform anastomosis. Usually, renal veins are intimately connected to each other inside and around the kidneys to form collateral circulation. Therefore, the effect of regional vein blockage is seemingly small.^1^ In the case of multiple renal veins, the frequency of out‐flow block by sacrifice of remaining veins is unknown, but a method to check for congestion by Doppler ultrasound examination has been reported.^2^

When transplanting the right kidney, it is unlikely that venous reconstruction will be necessary in most cases as the renal vein may be stretched due to its short length and blood flow in transplanted kidneys may be impaired. Most reconstructions often include end‐to‐side anastomosis with the external iliac vein. However, even if the external iliac vein is sufficiently dissected cranio‐caudally, the internal iliac vein must be amputated to move up the external iliac vein when the anastomosis is difficult due to the short length of the renal vein. Adversely, the internal iliac vein extends toward the pelvic wall and may cause a large amount of bleeding due to inadequate ligation.

If the renal vein still does not reach the external iliac vein, procedures for extending the vein by interposing various vessels have been reported. The reported interposing vessels are the saphenous vein in our cases,^3^, ^4^, ^5^ the gonadal vein,^6^, ^7^ using the renal vein of polycystic kidney removed concomitantly.^8^ SVGs have two shapes. One is a spiral vein graft in which the detubulized saphenous vein sutured in a spiral into a tubular shape, and the other is a cylindrical vein graft that split longitudinally and was sutured side‐to‐side into a tubular shape.^9^ At our hospital, we made a cylindrical vein graft because of its simplicity, which made it possible to form a Y‐shape in these cases, leading to successful transplantation. In these cases, although it was possible that one drainage vein was not sufficient to resolve the out‐flow block, two renal veins, anastomosed to the Y‐shaped SVG, may keep the transplanted kidney position stable and prevent extra tension on the corresponding vein.

## Conclusion

To our knowledge, this study is the first to report renal vein reconstruction using a Y‐shaped SVG. Our results indicate that the Y‐shaped SVG may be effective for planned venous reconstruction and treating anastomosis‐related complications. Although very few studies have addressed the reason behind anastomosis‐related complications in venous reconstruction, the Y‐shaped SVG is a seemingly effective method to treat such complications.

## Conflict of interest

The authors declare no conflict of interest.
